# Association of sleep variability and irregularity with gestational metabolic syndrome: a birth cohort study

**DOI:** 10.3389/fendo.2026.1736419

**Published:** 2026-05-13

**Authors:** Haiyan Gao, Libo Xu, Wenjuan Liu, Haibo Li, Bin Sun, Wei Li, Zhengqin Wu, Beihong Zheng, Yibing Zhu

**Affiliations:** 1 Fujian Maternity and Child Health Hospital, College of Clinical Medicine for Obstetrics & Gynecology and Pediatrics, Fujian Medical University, Fuzhou, China; 2 Fujian Obstetrics and Gynecology Hospital, Fuzhou, China; 3 Fujian Children’s Hospital, Fuzhou, China

**Keywords:** dyslipidemia, gestational metabolic syndrome, hyperglycemia, hypertension, irregularity, sleep, variability

## Abstract

**Background:**

Pregnant women exhibit a high prevalence of sleep disturbances; however, the relationships between sleep variability, sleep irregularity, and gestational metabolic syndrome (GMS) remain poorly understood. This study aimed to investigate prospective associations of sleep variability and irregularity with the odds of GMS and its individual components.

**Methods:**

Four sub-cohorts of pregnant women were established based on data from the Fujian Birth Cohort Study (FJBCS) collected from 2019 to 2021. Logistic regression models were adopted to assess the associations between sleep variability and irregularity during pregnancy and the occurrence of GMS and its components. Stratified analyses examined potential modification of effects by demographic factors. Restricted cubic spline (RCS) models were used to evaluate potential non-linear relationships.

**Results:**

Greater sleep variability was significantly associated with increased odds of GMS (aOR 1.348; 95% CI: 1.136-1.559) and dyslipidemia (aOR 1.086; 95% CI: 1.019-1.159), although no significant associations were found for hyperglycemia or hypertension. Sleep irregularity exhibited no significant association with GMS or its components. Subgroup analyses revealed increased vulnerability in women of advanced maternal age compared to younger age groups (aOR: 1.536 vs. 1.279). Those with a history of two or more prior pregnancies and natural pregnancies were more susceptible. RCS modeling confirmed the absence of nonlinear associations.

**Conclusions:**

Higher sleep variability is prospectively associated with an increased odds of GMS and dyslipidemia, with potential modulation by maternal age, conception method, and gravidity. No significant association was found between sleep irregularity and GMS or its components.

## 1 Introduction

Gestational metabolic syndrome (GMS) is a distinct pathological condition that manifests during pregnancy, characterized by a range of metabolic abnormalities, including pre-pregnancy overweight or obesity, dyslipidemia, impaired glucose homeostasis, and gestational hypertension. GMS constitutes a group of syndromes that have been associated with adverse pregnancy outcomes and long-term cardiovascular and metabolic risks for both mother and child (1, 2). Metabolic syndrome has been associated with a variety of adverse pregnancy outcomes, including but not limited to: miscarriage, preterm labor, preeclampsia, gestational diabetes mellitus (GDM), and stillbirth (3). A substantial surge in the prevalence of metabolic syndrome has been documented among women who are either currently pregnant or who are in the process of planning a pregnancy (3). Consequently, there has been a marked increase in recent years in interest in the prevention and screening of metabolic disorders in pregnancy, as well as in identifying potential risk factors to prevent the development of GMS. It is widely believed that sleeping habits constitute a potentially modifiable behavioral characteristic associated with metabolic health (4).

Current evidence suggests that sleep disorders are prevalent during pregnancy due to physical discomfort, psychological distress, and hormonal fluctuations (5). Sleep disorders have been shown to affect maternal and fetal health, leading to various mental and physical health issues (6). There is an increasing body of evidence suggesting that sleep disorders increase the incidence of metabolic syndrome in adult subjects (7, 8). However, the existing literature offers only preliminary insights into the relationship between sleep and GMS. Studies have shown that poor sleep quality during the early stages of pregnancy may increase the risk of elevated blood glucose levels and the likelihood of developing GDM in later stages of pregnancy (5). Besides, sleep apnea during pregnancy has been associated with an elevated risk of metabolic syndrome (9). Most studies on sleep during pregnancy have focused on sleep at a single point in time or average sleep duration, without tracking changes in sleep throughout pregnancy. It is widely recognized that pregnant women’s sleep patterns differ from those of non-pregnant women. This discrepancy is attributed to hormonal fluctuations and mood changes, which often result in significant variations in sleep patterns during pregnancy (10). Consequently, long-term monitoring of maternal sleep may inform future research on potential intervention strategies; however, interventional studies are needed to determine whether modifying sleep patterns improves maternal health outcomes.

Sleep variability refers to daily fluctuations in long-term sleep patterns, providing an additional dimension to average sleep duration (11). Sleep irregularity indicates the degree to which sleep patterns vary over time. Cross-sectional studies have demonstrated associations between irregular sleep patterns and a variety of physiological functions, including circadian rhythms, endocrinology, and metabolism (12). However, to date, there is no evidence on the association between sleep variability and sleep irregularity during pregnancy and GMS risk. A comprehensive evaluation is necessary to assess the impact of sleep variability and irregularity during pregnancy on GMS and its related components.

The Fujian Birth Cohort Study (FJBCS) is a prospective birth cohort study designed to monitor maternal and infant health during pregnancy. It collects comprehensive data, including sociodemographic characteristics, anthropometric and laboratory measurements, health-related behaviors, and health status. This study provides a foundation for further research on the relationship between sleep and GMS. The objective of this study was to evaluate the correlation between sleep variability and irregularity during pregnancy and the risk of GMS and its associated symptoms. The investigation utilized sleep data collected at various points throughout the pregnancy. Besides, stratified analyses were performed to examine the aforementioned associations across different populations.

## 2 Materials and methods

### 2.1 Study population

This prospective cohort study was based on the FJBCS, which is a large, comprehensive, and ongoing study designed to examine the effects of prenatal exposure on adverse pregnancy outcomes and the healthy development of children in Southeast China. The inclusion of pregnant women in this study commenced at their initial prenatal visit to the Fujian Provincial Maternity and Child Health Hospital and was contingent upon meeting the following criteria: 1) women between 18 and 45 years of age, 2) 8–13 weeks of gestation, 3) intention to undergo prenatal care and delivery at the Fujian Provincial Maternity and Child Health Hospital, and 4) written informed consent to participate in the study at the initial prenatal visit. Pregnant women with severe liver or kidney disease, serious cerebrovascular disease, serious psychiatric disorders, intellectual disability, or an inability to independently complete the study were excluded.

The present study was conducted on 13034 pregnant women who attended their initial prenatal health consultation and underwent preliminary antenatal examinations between March 2019 and December 2021. Of the participants, 325 pregnant women with multiple pregnancies and a complete absence of baseline metabolic syndrome-associated components were first excluded. A total of 12709 pregnant women who met the initial enrollment criteria were included for further sub-cohort construction.

The present study identified four sub-cohorts within the aforementioned population. The initial sub-cohort was conceived to investigate the longitudinal associations between sleep variability and irregularity and the incidence of GMS. The remaining three cohorts investigated the longitudinal associations between sleep variability and irregularity and the presence of GMS-related components. The selection process for participants in these four sub-cohorts, as well as the rationale behind the establishment of these sub-cohorts, is outlined below:

1) Participants lacking essential data to define GMS at baseline (n = 1207) and follow-up endpoints (n = 1515) were excluded from the study, and those diagnosed with GMS at baseline (n = 17) were further excluded. In the final analysis of sub-cohort 1, 9970 participants without a previous diagnosis of GMS were included. 2) The second sub-cohort was designed to examine longitudinal associations between sleep variability and irregularity and the development of hypertension. Participants who lacked the necessary information to define hypertension at baseline (n = 95) and follow-up endpoints (n = 1391) were excluded from the study. Besides, participants who were diagnosed with hypertension at baseline (n = 141) were excluded from further analysis. The final sample comprised 11, 082 participants without pre-existing hypertension. 3) The third sub-cohort was designed to examine longitudinal associations between sleep variability and irregularity and the development of hyperglycemia. Participants lacking the necessary baseline data for hyperglycemia assessment (n = 1101) and those with subsequent follow-up (n=679) were excluded from the study, along with those diagnosed with hyperglycemia at baseline (n = 1536). The final sample comprised 9393 participants without pre-existing hyperglycemia. 4) The fourth sub-cohort was designed to examine the longitudinal association between sleep variability, as well as irregularity, and the occurrence of dyslipidemia. Participants who lacked the information needed to define dyslipidemia at baseline (n = 949) and follow-up endpoints were excluded (n = 688), and those diagnosed with dyslipidemia at baseline were further excluded (n = 78). Finally, 10,994 participants without a diagnosis of dyslipidemia were included in the final analysis. The study flow chart is presented in **Supplementary Figure 1**.

### 2.2 Ethics

The studies involving human participants in the FJBCS were reviewed and approved by the Research Ethics Committee of Fujian Maternity and Child Health Hospital (approval number: 2019-200-06). Informed consent was obtained from all participants before their involvement in the study. The research methods and processes adhered to the ethical principles set out in the Declaration of Helsinki for medical research and to local statutory requirements. The study protocol explicitly guaranteed participants the right to withdraw their consent at any time, ensuring that such a decision would not result in adverse consequences.

### 2.3 Exposure assessment

The assessment of sleep parameters was conducted using the Pittsburgh Sleep Quality Index (PSQI) questionnaire (13), which is a self-reported measure of sleep quality. The PSQI items were obtained through self-reporting by pregnant women regarding their overall sleep over the previous month. These reports included bedtime, wake time, sleep duration, and other relevant indicators. Sleep duration was calculated from the subject’s self-reported habitual bedtime, wake time, and duration of falling asleep (14). Sleep midpoint (midpoint between sleep start and sleep end) was then calculated as: sleep onset time + [(wake-up time-sleep onset time)/2] (15). In line with the conceptual framework established in previous studies, we identified two distinct dimensions of sleep pattern instability. Sleep variability was defined as the standard deviation (SD) of sleep duration across trimesters, reflecting fluctuations in sleep quantity. Sleep irregularity was defined as the SD of sleep midpoint across trimesters, reflecting day-to-day deviations in sleep timing (i.e. circadian irregularity). While both metrics use SD across trimesters, they represent different aspects of sleep health: sleep variability relates to consistency in sleep duration, whereas sleep irregularity relates to consistency in sleep–wake timing. This distinction is supported by prior literature (16). PSQI data were collected at three time points: during the first (11-13^+6^ weeks), second (22-26^+6^ weeks), and third (32-36^+6^ weeks) trimesters. Sleep variability was defined as the intra−individual standard deviation of self−reported sleep duration across the three trimesters. Sleep irregularity was defined as the intra−individual standard deviation of sleep midpoint across the three trimesters. These values were derived from the PSQI self-report questionnaire completed by each individual at each trimester.

### 2.4 Outcome assessment

Anthropometric and biochemical measurements were obtained during the first, second, and third trimesters and at delivery. These measurements included height, weight, blood pressure, fasting plasma glucose (FPG), and triglycerides (TG). The primary outcome of interest in this study was GMS and its related components at delivery. Blood pressure was measured using a non-invasive oscillometric upper-arm device in a seated position. FPG and TG levels were assessed using latex-enhanced immunoassays and enzymatic procedures on automated analyzers after a minimum fasting period of 8 hours. The diagnosis of GMS was established according to the diagnostic criteria for metabolic syndrome, as developed by the Chinese Medical Association Diabetes Society in 2004, with subsequent refinements adapted from Hui Wu et al. (17). The diagnostic criteria for GMS in this study were as follows: 1) Pre-pregnancy BMI ≥ 25 kg/m^2^, suggesting overweight or obesity (derived from early pregnancy records). 2) Hyperglycemia: FPG ≥ 5.1 mmol/L (measured at delivery). 3) Hypertension: Defined as blood pressure ≥ 140/90 mmHg (measured at delivery). 4) Dyslipidemia: Characterized by TG levels ≥ 3.23 mmol/L (measured at delivery). GMS diagnosis was made when three or all of the aforementioned criteria were met. Although pre-pregnancy BMI reflects baseline metabolic status rather than gestational changes, established literature supports its inclusion as an integral component of gestational metabolic syndrome (17). Pre-pregnancy adiposity establishes a metabolic baseline that interacts with *de novo* gestational metabolic disturbances. Therefore, including pre-pregnancy BMI in the GMS definition captures both pre-existing and gestational metabolic risks.

### 2.5 Covariates

Covariates were selected based on clinical and epidemiological relevance, as identified from prior literature and a directed acyclic graph (DAG), encompassing maternal age (years), pre-pregnancy BMI (underweight, normal weight, overweight or obese), ethnicity (han or minority), educational levels (junior high school and below, high school, undergraduate degree and graduate and above), urban-residence (yes or no), gravidity (1, ≥2), parity (0, 1, ≥ 2), assisted reproduction (yes or no), smoking status (yes or no), alcohol consumption status (yes or no), coffee consumption status (yes or no), tea consumption status (yes or no), average outdoor time (< 30, 30–60, 60–120, and ≥ 120min), pre-pregnancy diabetes (yes or no), pre-pregnancy hypertension (yes or no), prior abnormal pregnancy (yes or no), prior pregnancy complications (yes or no), prior GH (previous history of gestational hypertension) (yes or no) and prior GDM (previous history of gestational diabetes) (yes or no).

### 2.6 Statistical analysis

Descriptive statistics were presented as the mean ± standard deviation for continuous variables exhibiting a normal distribution. Frequency and percentage were utilized for categorical variables. Mean, standard deviation, and interquartile range were calculated for variability in sleep duration and sleep midpoint.

Multivariable unconditional logistic regression models were used to estimate the association between sleep variability or sleep irregularity and the subsequent odds of GMS and its components. Sleep variability and sleep irregularity were analyzed as continuous variables. A range of models is employed to assess the robustness of the estimates. Model 1 constituted a univariate analysis, whereas model 2 included covariates for maternal age, ethnicity, educational levels, and urban residence. Finally, in addition to adjusting for all covariates in model 2, model 3 was further adjusted for gravidity, parity, assisted reproduction, smoking status, alcohol consumption status, coffee consumption status, tea consumption status, average outdoor time, pre-pregnancy diabetes, pre-pregnancy hypertension, prior abnormal pregnancy, prior pregnancy complications, prior GH, and prior GDM. To assess potential selection bias arising from missing outcome data, we performed a sensitivity analysis using inverse probability weighting. The adjusted variables were consistent with those in Model 3. To complement the continuous analyses, we categorized sleep variability measures into quartiles based on their distribution in the study population. Multivariable logistic regression models were fitted with the lowest quartile as the reference category, and tests of linear trend were conducted by including the median of each quartile as a continuous term in the models.

Subgroup analyses stratified by maternal age (< 35, ≥ 35), assisted reproduction (yes or no), and gravidity (1, ≥ 2) were pre−specified based on their clinical relevance and potential to modify the association between sleep variability or sleep irregularity and the subsequent odds of GMS and its components. Given that pre-pregnancy BMI is a component of the GMS definition, it was not included in the primary regression models to avoid over-adjustment. Potential effect modification by maternal age, conception method, and gravidity was assessed by including interaction terms (sleep variability/irregularity × each modifier) in the multivariable models and evaluating their significance using likelihood ratio tests.

To assess the robustness of the logistic regression model after adjustment, we further explored the dose-response relationship between sleep variability or sleep irregularity and the occurrence of GMS and its components by fitting a multivariable logistic regression model using restricted cubic spline (RCS). To flexibly model potential non-linear relationships, we applied restricted cubic splines with 3 knots placed at the 10^th^, 50^th^, and 90^th^ percentiles of the exposure distribution. The number of knots was selected based on the Akaike Information Criterion to balance model fit and parsimony, and the final model corresponded to the lowest Akaike information criterion (AIC) value.

To assess whether exclusions due to missing data or prevalent conditions at baseline introduced selection bias, we compared baseline characteristics between participants included in each sub-cohort and those excluded. The variables compared included maternal age, ethnicity, education level, urban residence, and pre-pregnancy BMI. Continuous variables were compared using independent samples t-tests or Mann-Whitney U tests, as appropriate based on distribution, and categorical variables were compared using chi-square tests or Fisher’s exact tests.

The proportion of missing values for major confounders was well below 5%; therefore, missing values were not included. Two-sided *p* < 0.05 was considered statistically significant. Statistical analyses were performed with R software (Version 4.3.3, Vienna, Austria).

## 3 Results

### 3.1 Descriptive characteristics

**Table 1** presents a summary of the demographic characteristics across the four sub-cohorts. Nearly all participants were of Han ethnicity (98.1%), and the majority had completed an undergraduate education, with rates of 71.6%, 71.7%, 72.5%, and 71.8% observed across the groups. The mean maternal age was consistently around 30 years. With respect to pre-pregnancy body mass index, most women fell within the normal range, as reflected by proportions of 75.5%, 75.3%, 75.2%, and 75.1% in the respective sub-cohorts. Regarding adverse outcomes, the incidence of gestational hypertension, hyperglycemia, dyslipidemia, and metabolic syndrome was 289 (2.9%), 324 (2.9%), 3283 (35.0%), and 5,335 (48.5%) cases across the four groups, respectively. The mean (SD) sleep variability was 0.87 (0.66) hours, with an IQR of 0.45–1.12 hours. For sleep irregularity, the mean (SD) was 0.51 (0.39) hours, with an IQR of 0.20–0.71 hours (**Supplementary Table 1**). As shown in **Supplementary Table 2**, in sub-cohort 1 and sub-cohort 4, no significant differences were observed between included and excluded participants for any baseline variable (all *p* > 0.05). In sub-cohort 2 and sub-cohort 3, pre-pregnancy BMI differed significantly between included and excluded participants (*p* < 0.05). No significant differences were observed for other baseline variables.

**Table 1:** Maternal characteristics of the study population in four sub-cohorts.

Variables	Sub-Cohort 1	Sub-Cohort 2	Sub-Cohort 3	Sub-Cohort 4
**No. of participants, n**	9970	11082	9393	10994
**Maternal age(years) ± mean (SD)**	30.5 ± 4.0	30.5 ± 4.0	30.4 ± 3.9	30.6 ± 4.0
Maternal age				
<35 years	8604 (86.3)	9531 (86.0)	8129 (86.5)	9448 (85.9)
≥35 years	1366 (13.7)	1551 (14.0)	1264 (13.5)	1546 (14.1)
**Ethnicity-Han, n (%)**	9970 (98.1)	10872 (98.1)	9218 (98.1)	10784 (98.1)
Education, n (%)^a^				
Junior high school and below	831 (8.4)	902 (8.1)	731 (7.7)	911 (8.3)
High School	1272 (12.8)	1415 (12.8)	1440 (12.1)	1380 (12.6)
Undergraduate degree	7143 (71.6)	7951 (71.7)	6813 (72.5)	7890 (71.8)
Graduate and above	713 (7.2)	803 (7.2)	701 (7.5)	802 (7.3)
**Urban-residence, n (%)** ^a^	7228 (72.5)	8021 (72.4)	6878 (73.2)	8115 (73.8)
Gravidity, n(%)				
1	4230 (42.4)	4663 (42.1)	4163(44.3)	4738(43.1)
≥2	5740 (57.6)	6419 (57.9)	5230 (55.7)	6256 (56.9)
Parity, n(%)				
0	5782 (58.0)	6436 (58.1)	5623 (59.9)	6488 (59.0)
1	3775 (37.9)	4188 (37.8)	3400 (36.2)	4061 (36.9)
≥2	413 (4.1)	458 (4.1)	370 (3.9)	445 (4.0)
**Assisted reproduction, n (%)**	647 (6.5)	747 (6.7)	627 (6.7)	771 (7.0)
Pre-pregnancy BMI (kg/m^2^), n (%)				
Normal weight	7528 (75.5)	8348 (75.3)	7059 (75.2)	8257 (75.1)
Underweight	1611 (16.2)	1800 (16.2)	1581 (16.8)	1761 (16.0)
Overweight+ Obese	831 (8.3)	899 (8.1)	721 (7.7)	940 (8.6)
**Pre-pregnancy diabetes, n (%)**	17 (0.2)	25 (0.2)	10 (0.1)	20 (0.2)
**Pre-pregnancy hypertension, n (%)**	11 (0.1)	8 (0.1)	7 (0.1)	12 (0.1)
**Prior abnormal pregnancy, n (%)**	3534 (35.4)	4003 (36.1)	3242 (34.5)	3895 (35.4)
**Prior Pregnancy Complications, n (%)**	746 (7.5)	830 (7.5)	611 (6.5)	810 (7.4)
**Prior GH, n (%)**	100 (1.0)	109 (1.0)	85 (0.9)	109 (1.0)
**Prior GDM, n (%)**	384 (3.9)	427 (3.9)	295 (3.1)	421 (3.8)
**Smoking before pregnancy, n (%)**	192 (1.9)	211 (1.9)	181 (1.9)	209 (1.9)
**Drinking alcohol before pregnancy, n (%)**	1205 (12.1)	1325 (12.0)	1111 (11.8)	1280 (11.6)
**Drinking coffee before pregnancy, n (%)** ^a^	2448 (24.6)	2698 (24.3)	2265 (24.1)	2667 (24.3)
**Drinking tea before pregnancy, n (%)** ^a^	3284 (32.9)	3633 (32.8)	3076 (32.7)	3548 (32.3)
Average outhoor time , n (%)^a^				
<30min	5998 (60.2)	6682 (60.3)	5649 (60.1)	6613 (60.2)
30-60min	2956 (29.6)	3270 (29.5)	2804 (29.9)	3286 (29.9)
60-120min	765 (7.7)	852 (7.7)	711 (7.6)	834 (7.6)
≥120min	203 (2.0)	228 (2.1)	184 (2.0)	212 (1.9)
Outcome variables				
GMS, n (%)	289 (2.9)	—	—	—
Hypertension, n (%)	—	324 (2.9)	—	—
Hyperglycemia, n (%)	—	—	3283 (35.0)	—
Dyslipidemia, n (%)	—	—	—	5335 (48.5)

GMS, gestational metabolic syndrome; prior GDM, previous history of gestational diabetes; prior GH, previous

^a^ Represents missing values.

### 3.2 Associations of sleep variability and sleep irregularity with GMS and its components

**Table 2** provides an overview of the relationship between sleep variability and irregularity, as measured by the GMS and its components. Among pregnant women who did not have GMS at the beginning of the study, the univariate analysis revealed a significant association between variations in sleep and the subsequent development of GMS (aOR, 1.243; 95% CI: 1.068-1.448), as shown in model 1. In model 2, the association remained statistically significant after adjusting for demographic characteristics (aOR, 1.328; 95% CI: 1.123-1.570), and the aOR increased. Model 3 exhibited an association that remained statistically significant (aOR, 1.348; 95%CI: 1.136-1.599) after further adjustment for other confounders based on model 2. The results for model 3 revealed that for every one-hour increase in sleep variability, the odds of GMS increased by 34.8%. For pregnant women without dyslipidemia at baseline, sleep variability was significantly associated with dyslipidemia, and aOR did not fluctuate much after adjusting for different confounders. In model 3, for every 1-hour increase in sleep variability, the odds of dyslipidemia increased by 8.6% (aOR, 1.086; 95% CI: 1.019-1.159) (**Table 2**). Conversely, for pregnant women without hypertension and hyperglycemia at baseline, no significant associations were found between sleep variability and these outcomes. Moreover, no significant associations were found between sleep irregularity and GMS, hypertension, hyperglycemia, or dyslipidemia across the three models. The inverse probability weighting sensitivity analyses showed that, even in sub-cohorts 2 and 3, after adjusting for potential selection bias, the direction, magnitude, and statistical significance of the associations between sleep variability/irregularity and hypertension/hyperglycemia remained consistent with the primary analyses (**Supplementary Table 3**). This suggests that despite the observed difference in pre-pregnancy BMI, selection bias yielded a limited impact on the main conclusions. Compared with women in the lowest quartile of sleep variability, those in the highest quartile exhibited significantly increased odds of GMS (aOR = 1.522, 95% CI: 1.034–2.241), with a significant linear trend across quartiles (*p*−trend = 0.027). Similar patterns were observed for sleep midpoint variability and for the individual metabolic components (**Table 3**).

**Table 2:** Associations of sleep variability and sleep irregularity with GMS and its components.

Variables	Model 1		Model 2^a^		Model 3^b^	
	cOR (95% CI)	*P*	aOR (95% CI)	*P*	aOR (95% CI)	*P*
Sub-Cohort 1 Occurrence of GMS (N = 9970)						
sleep variability	1.243(1.068-1.448)	**<0.005**	1.328(1.123-1.570)	**<0.001**	1.348(1.136-1.599)	**<0.001**
sleep irregularity	1.170(0.882-1.551)	0.276	1.061(0.766-1.471)	0.721	1.062(0.773-1.459)	0.711
Sub-Cohort 2 Occurrence of Hypertension (N = 11082)						
sleep variability	1.056(0.901-1.238)	0.501	1.073(0.896-1.284)	0.444	1.081(0.901-1.297)	0.403
sleep irregularity	1.011(0.758-1.348)	0.943	1.102(0.804-1.510)	0.547	1.102(0.800-1.517)	0.552
Sub-Cohort 3 Occurrence of Hyperglycemia (N = 9393)						
sleep variability	1.018(0.954-1.086)	0.586	1.028(0.955-1.106)	0.467	1.020(0.947-1.098)	0.600
sleep irregularity	1.057(0.948-1.178)	0.319	1.015(0.898-1.148)	0.811	1.008(0.891-1.144)	0.897
Sub-Cohort 4 Occurrence of Dyslipidemia (N = 10994)						
sleep variability	1.065(1.006-1.127)	**0.029**	1.086(1.019-1.158)	**0.011**	1.086(1.019-1.159)	**0.012**
sleep irregularity	1.077(0.978-1.186)	0.131	1.074(0.964-1.196)	0.197	1.069(0.959-1.193)	0.228

GMS, gestational metabolic syndrome; prior GDM, previous history of gestational diabetes; prior GH, previous history of gestational hypertension; aOR, adjusted relative risk; CI, confidence interval; cOR, crude relative risk.

^a^ aOR and 95% CI estimated with logistics regression adjusted for maternal age, ethnicity, educational levels and urban-residence in model 2.

^b^ Model 3 further adjust, gravidity, parity, assisted reproduction, smoking status, alcohol consumption status, coffee consumption status, tea consumption status, average outdoor time, pre-pregnancy diabetes, pre-pregnancy hypertension, prior abnormal pregnancy, prior pregnancy complications, prior GH and prior GDM.

Bold values indicate p<0.05.

**Table 3:** Associations of sleep variability quartiles and sleep irregularity quartiles with GMS and its components.

Variables	Q1 (lowest)	Q2	Q3	Q4 (highest)	*P* for trend
		aOR (95% CI)^a^	aOR (95% CI)^a^	aOR (95% CI)^a^	
Sleep variability					
GMS	1.000 (ref)	1.091(0.724-1.643)	1.446(0.985-2.124)	1.522(1.034-2.241)	**0.027**
Hypertension	1.000 (ref)	1.109(0.777-1.582)	1.102(0.772-1.573)	1.159(0.811-1.658)	0.744
Hyperglycemia	1.000 (ref)	0.994(0.868-1.138)	0.936(0.817-1.071)	0.983(0.859-1.126)	0.358
Dyslipidemia	1.000 (ref)	1.056(0.937-1.190)	1.104(0.980-1.243)	1.126(0.998-1.269)	0.065
Sleep irregularity					
GMS	1.000 (ref)	0.808(0.556-1.173)	1.037(0.722-1.490)	0.932(0.639-1.359)	0.573
Hypertension	1.000 (ref)	0.921(0.650-1.303)	1.063(0.751-1.504)	1.015(0.712-1.448)	1.000
Hyperglycemia	1.000 (ref)	0.976(0.855-1.114)	1.052(0.918-1.204)	1.028(0.896-1.179)	0.266
Dyslipidemia	1.000 (ref)	0.964(0.859-1.083)	1.081(0.959-1.219)	1.054(0.934-1.189)	0.174

GMS, gestational metabolic syndrome; prior GDM, previous history of gestational diabetes; prior GH, previous history of gestational hypertension.

^a^ 4aOR, adjusted relative risk; CI, confidence interval. aOR and 95% CI estimated with logistics regression adjusted for maternal age, ethnicity, educational levels, urban-residence, gravidity, parity, assisted reproduction, smoking status, alcohol consumption status, coffee consumption status, tea consumption status, average outdoor time, pre-pregnancy diabetes, pre-pregnancy hypertension, prior abnormal pregnancy, prior pregnancy complications, prior GH and prior GDM.

Bold values indicate p<0.05.

### 3.3 Associations of sleep variability and sleep irregularity with GMS and its components by basic characteristics.

**Figure 1:**
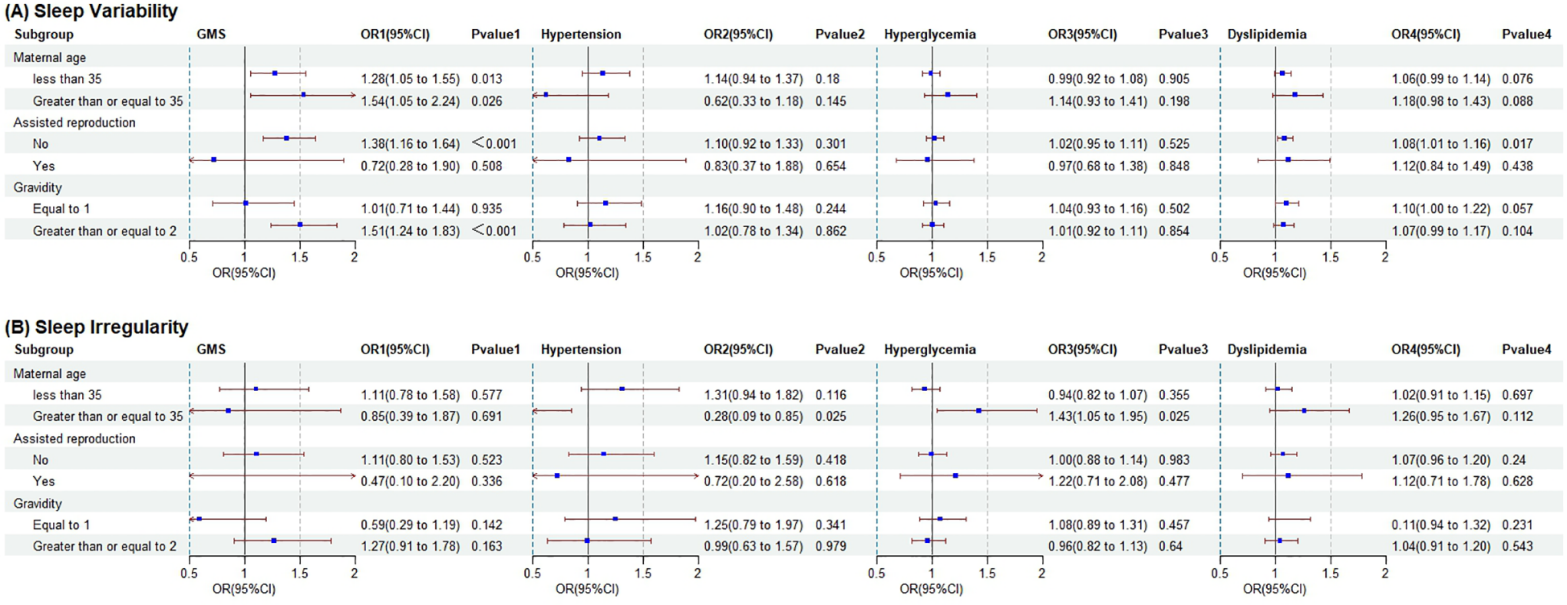
**(A)** Sleep variability and **(B)** Sleep irregularity. Subgroup analyses of the association between sleep variability/irregularity and GMS and its components. Forest plots showing aOR and 95% CI for the association between sleep variability/irregularity and GMS and its components, stratified by maternal age, conception method and gravidity. The results suggested that these associations may vary according to maternal age, conception method and gravidity. GMS, gestational metabolic syndrome; prior GDM, previous history of gestational diabetes; prior GH, previous history of gestational hypertension; aOR, adjusted relative risk; CI, confidence interval; cOR, crude relative risk. aOR and 95% CI estimated with logistics regression adjusted for maternal age, ethnicity, educational levels, urban-residence, gravidity, parity, assisted reproduction, smoking status, alcohol consumption status, coffee consumption status, tea consumption status, average outdoor time, pre-pregnancy diabetes, pre-pregnancy hypertension, prior abnormal pregnancy, prior pregnancy complications, prior GH and prior GDM.

**Figure 1** illustrates the associations of sleep variability and sleep irregularity with GMS and its individual components, stratified across various demographic and clinical subgroups. The four sub-cohorts were then analyzed in groups based on maternal age, assisted reproduction, and gravidity. As demonstrated in **Figure 1A**, in sub-cohort 1, the degree of association between sleep variability and GMS varied across pregnant women in distinct maternal age groups. A higher odds ratio of 1.536 was observed among pregnant women of advanced age compared with those of non-advanced age (1.279). During stratification by mode of conception, the correlation between sleep variability and GMS was statistically significant only in the natural pregnancy population, with an aOR of 1.384 (95% CI: 1.165-1.645). Following stratification by gravidity, a statistically significant association between sleep variability and GMS was observed only in the stratum comprising women with gravidity ≥2, with an aOR of 1.507 (95% CI 1.240-1.832). Sleep irregularity and GMS remained statistically nonsignificant across strata (**Figure 1B**).

In sub-cohort 2, subgroup analyses were performed according to maternal age, assisted reproduction, and gravidity. These analyses revealed no significant associations between sleep variability (**Figure 1A**) and sleep irregularity (**Figure 1B**) with hypertension. In sub-cohort 3, a significant association was identified between sleep irregularity and hyperglycemia, specifically among pregnant women of advanced age. This finding yielded a corresponding aOR of 1.430 (95% CI: 1.047-1.954) (**Figure 1B**). No significant associations were observed in the other strata. No significant association between sleep variability and hyperglycemia was identified across strata (**Figure 1B**). In sub-cohort 4, a significant association between sleep variability and dyslipidemia was identified in the naturally pregnant population when stratified by mode of conception, with an aOR of 1.084 (95% CI: 1.015-1.158) (**Figure 1A**). However, no significant associations were observed in the other strata. Similarly, no significant correlation was identified between sleep irregularity and dyslipidemia across the various factor strata (**Figure 1B**).

Formal interaction tests revealed that the associations between sleep variability and outcomes were significantly modified by maternal age (*p* for interaction < 0.001; *p* for interaction = 0.002), conception method (*p* for interaction = 0.002; *p* for interaction = 0.023), and gravidity (*p* for interaction < 0.001; *p* for interaction = 0.013) in sub-cohort 1 and sub-cohort 4, while in others (sub-cohort 2 and sub-cohort 3) the evidence for effect modification was limited or absent. Detailed interaction *p*−values are provided in **Supplementary Table 4**.

### 3.4 Dose-response relationships of sleep variability and sleep irregularity with GMS and its components

**Figure 2:**
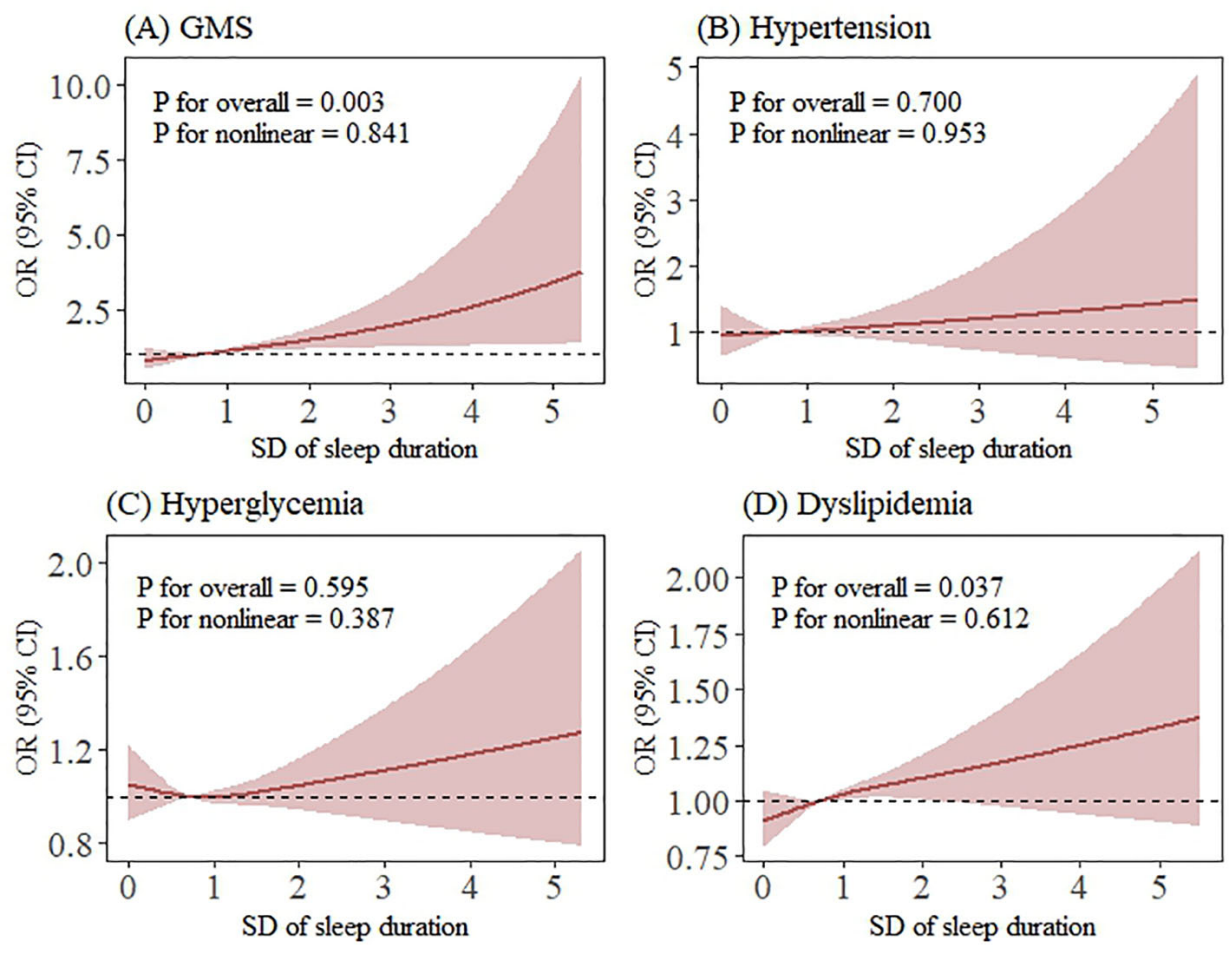
Restricted cubic spline analysis of the association between sleep variability and GMS and its components (smoothing by RCS function with three knots). Dose-response relationships were observed between sleep variability and **(A)** GMS, **(B)** hypertension, **(C)** hyperglycaemia and **(D)** dyslipidaemia. aOR and 95% CI (shaded areas) are shown. RCS analysis revealed no significant nonlinear associations of sleep variability with GMS or its components. Abbreviations: GMS, gestational metabolic syndrome; prior GDM, previous history of gestational diabetes; prior GH, previous history of gestational hypertension. **(A)** Developing to GMS, **(B)** Developing to Hypertension, **(C)** Developing to Hyperglycemia, **(D)** Developing to Dyslipidemia. All models were adjusted for maternal age, ethnicity, educational levels, urban-residence, gravidity, parity, assisted reproduction, smoking status, alcohol consumption status, coffee consumption status, tea consumption status, average outdoor time, pre-pregnancy diabetes, pre-pregnancy hypertension, prior abnormal pregnancy, prior pregnancy complications, prior GH and prior GDM.

**Figure 3:**
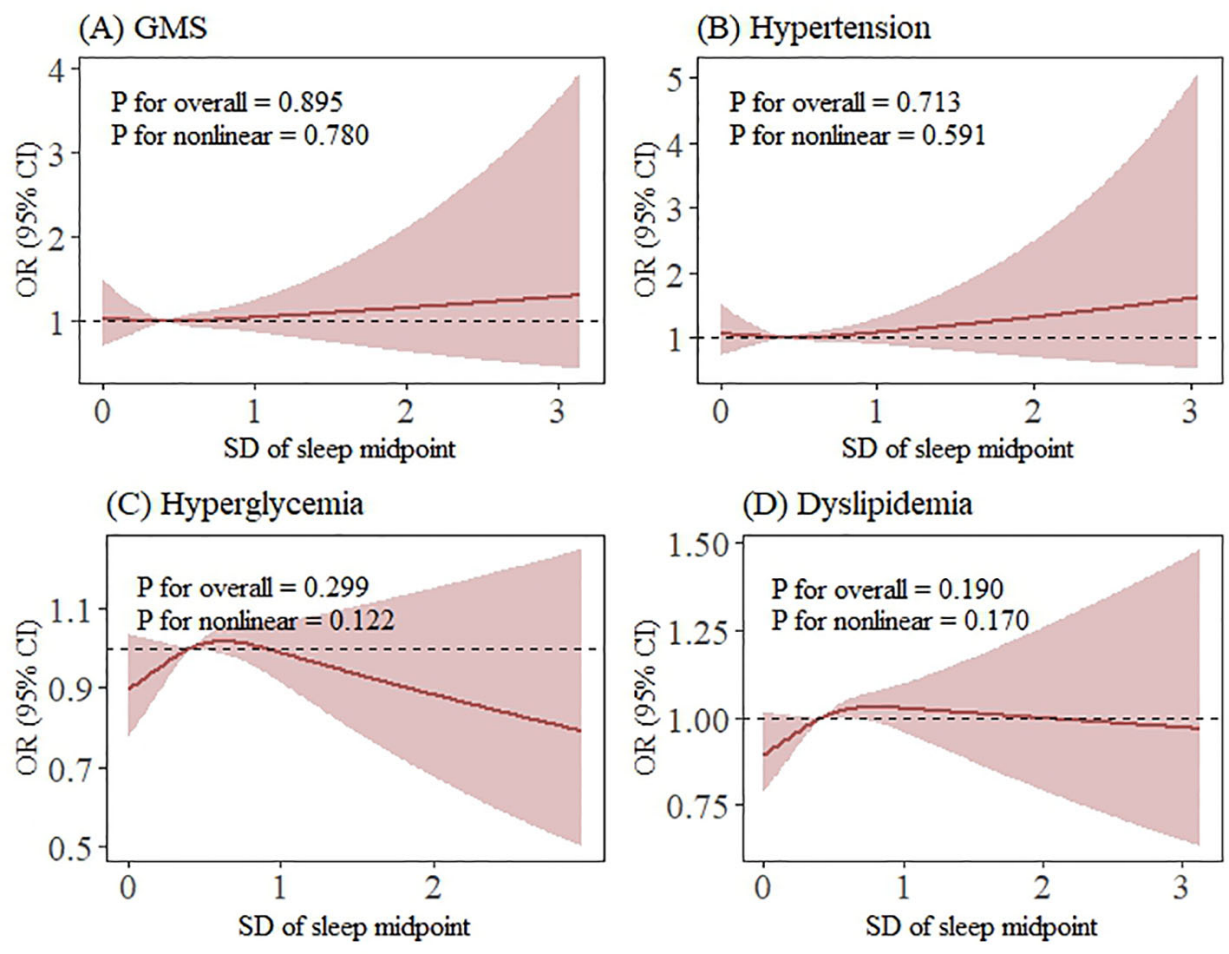
Restricted cubic spline analysis of the association between sleep irregularity and GMS and its components (smoothing by RCS function with three knots). Dose-response relationships were observed between sleep irregularity and **(A)** GMS, **(B)** hypertension, **(C)** hyperglycaemia and **(D)** dyslipidaemia. aOR and 95% CI (shaded areas) are shown. RCS analysis revealed no significant nonlinear associations of sleep irregularity with GMS or its components. Abbreviations: GMS, gestational metabolic syndrome; prior GDM, previous history of gestational diabetes; prior GH, previous history of gestational hypertension. **(A)** Developing to GMS, **(B)** Developing to Hypertension, **(C)** Developing to Hyperglycemia, **(D)** Developing to Dyslipidemia. All models were adjusted for maternal age, ethnicity, educational levels, urban-residence, gravidity, parity, assisted reproduction, smoking status, alcohol consumption status, coffee consumption status, tea consumption status, average outdoor time, pre-pregnancy diabetes, pre-pregnancy hypertension, prior abnormal pregnancy, prior pregnancy complications, prior GH and prior GDM.

As illustrated in **Figure 2**, **3**, the RCS analysis revealed no significant non-linear associations of sleep variability and irregularity with GMS or its individual components. However, the association of sleep variability with GMS and dyslipidemia was further confirmed in the RCS model (*p* for overall = 0.003; *p* for overall = 0.037), and the estimated curves exhibited a monotonically increasing linear dose-response relationship (*p* for nonlinear = 0.841; *p* for nonlinear = 0.612) (**Figures 2A, D**).

## 4 Discussion

Our findings indicated that sleep variability is a key correlate of metabolic health in pregnancy, with each 1-hour increase in sleep deviation associated with a 35% and 9% increase in the odds of GMS and dyslipidemia, respectively. Although these effect sizes are modest and the study design is observational, the widespread nature of irregular sleep among pregnant women suggests significant public health implications. To contextualize these findings, we examined the distribution of sleep variability in our cohort. The IQR for sleep variability was 0.41–1.12 hours, suggesting that a 1−hour difference lies near the upper bound of the IQR and is experienced by a substantial proportion of pregnant women. Thus, the effect sizes reported in the present study (35% higher odds of GMS per 1−hour increase in sleep variability) are relevant to clinically observable differences in sleep patterns during pregnancy, not merely to extreme outliers. To facilitate clinical interpretation, we present the quartile analysis. Compared with women in the lowest quartile of sleep variability, those in the highest quartile had significantly higher odds of GMS. There was also a significant linear trend across the quartiles. These quartile-based contrasts complement the per-hour increase estimates. Moreover, no significant association was found between sleep irregularity and GMS or its components. Taken together, these findings suggest that sleep variability may play a crucial role in metabolic health during pregnancy, independent of sociodemographic factors, lifestyle habits, and prior disease history. Besides, the overall incidence of GMS in this cohort was 2.9% (n=289). According to a Japanese survey of adults aged 20–40 years, the probability of metabolic syndrome per 1000 persons per year was 2.2, 5.5, and 10.2 for women aged 20, 30, and 40 years, respectively (18). This higher prevalence likely reflects differences in the study populations: the Japanese cohort comprised the general female population, whereas our study focused on pregnant women, whose unique metabolic challenges may exacerbate underlying predispositions to metabolic dysfunction (19). In a study conducted by Fentie et al., the prevalence of metabolic syndrome was reported to be 3.14%, 4.72%, and 5.35% in early, mid, and late pregnancy, respectively (20). The lower prevalence of GMS observed in our study likely reflects our exclusion of baseline GMS cases and focus on new-onset cases within one year, as well as differences in diagnostic criteria, sample sizes, and laboratory methods.

Over the years, a limited number of previous studies have revealed an association between sleep and GMS. A cross-sectional study of 318 pregnant women in Ethiopia found that unhealthy sleep duration was associated with an increased odds of GMS (aOR = 5.6; 95% CI: 2.4, 13.1) (20). A study by Facco FL et al. found that an apnoeic hypoventilation index during pregnancy was associated with an increased odds of metabolic syndrome (aOR = 2.46; 95%CI:1.59-3.76) (9). This study builds upon existing research identifying sleep-related genetic polymorphisms, specifically within circadian clock and fat taste perception pathways, that contribute to obesity and metabolic dysregulation during pregnancy (21). While previous studies have established these associations, they have largely relied on cross-sectional designs that capture sleep patterns at a single time point. The present study addresses this limitation by utilizing the prospective FJBCS dataset, which tracks sleep measurements longitudinally across all three trimesters. Besides, the relationship between sleep variability and irregularity during pregnancy and GMS was assessed. Importantly, sleep variability during pregnancy was associated with an elevated risk of GMS. While our study substantiates that suboptimal sleep during pregnancy adversely affects metabolic health, we undertook an exploratory analysis to determine whether this association varied by maternal age, mode of pregnancy, and gravidity.

Subgroup analyses indicated that this association was related to maternal age, mode of pregnancy, and gravidity. Concurrently, the RCS results further corroborated the aforementioned findings. Consistent with the literature (22), our results demonstrated that women aged ≥35 years exhibit greater susceptibility to metabolic complications, thereby further validating our primary hypothesis. This finding aligned with the results of the present study, thereby substantiating the primary hypothesis. A study of 126 women (62 assisted conception, 64 spontaneous) followed for 6–12 weeks postpartum found a higher prevalence of metabolic syndrome in the assisted conception group, though the difference did not reach statistical significance. Larger sample sizes and extended follow-up are needed to validate these findings (23). In the present study, the smaller sample size of the assisted reproductive cohort (n = 647) compared with the natural pregnancy cohort (n = 9,323) likely limited the statistical power to detect a significant association between sleep variability and GMS. This potential association remains a focus of our ongoing birth cohort study, which features an expanded assisted reproduction population. Consistently, Akter et al. conducted a cross-sectional study that found a positive correlation between gravidity and parity and the prevalence of metabolic syndrome (24). To further explore these relationships, subgroup analyses were pre-specified based on prior literature. Although we observed significant interactions in some sub−cohorts, particularly for Sub-Cohort 1 and Sub-Cohort 4, these findings should be interpreted with caution, given the multiple comparisons performed and the exploratory nature of the subgroup analyses. The inconsistency across sub−cohorts may reflect true biological differences or chance. Although we did not formally adjust for multiple testing, the consistency of the associations across strata and outcomes reduces the likelihood that the results are solely due to chance. Subgroup analyses indicated the possibility of differences in associations according to maternal age, conception method, and parity for certain outcomes. However, given the exploratory nature of these analyses and the multiple comparisons performed, these findings should be treated as hypothesis-generating rather than hypothesis-confirming. Further research involving larger sample sizes and pre-specified subgroup hypotheses is required to establish whether these factors modify the relationship between sleep variability and gestational metabolic outcomes.

The extant research related to sleep variability, sleep irregularity, and metabolic health is relatively limited and has primarily been conducted in the form of cross-sectional studies. A multi-ethnic study of atherosclerosis, encompassing 6814 men and women aged 45–84 years, found that greater variability in sleep duration is associated with a higher incidence of metabolic abnormalities (25). A cross-sectional study of 3880 middle-aged and older adults, with a mean age of 55 to 58 years, revealed a significant association between sleep irregularity and metabolic syndrome (26). In adolescents, the presence of circadian sleep disorders has been demonstrated to exacerbate visceral obesity, thereby increasing the likelihood of developing metabolic syndrome (27). While most studies on sleep variability address middle-aged or adolescent cohorts, our prospective research extends this evidence to pregnant women. We identified a correlation between sleep variability and increased odds of dyslipidemia; however, no associations were found with hypertension, hyperglycemia, or sleep irregularity. This study represents the first to specifically explore the link between sleep variability and hyperglycemia in this population. Some epidemiological studies have shown that circadian rhythm disorders, sleep deprivation, and shift work are associated with an elevated risk of dyslipidemia (28). However, another study found no significant association between sleep duration and the risk of dyslipidemia (29). A review of epidemiological studies from 2014 to 2019 found that variability in sleep duration was not significantly associated with hypertension risk. In contrast, sleep irregularity was more strongly associated with blood pressure and the risk of hypertension. The authors hypothesized that this association may be mediated by changes in insulin sensitivity and inflammation (30). However, the present study did not yield analogous findings. Zuraikat FM et al. provided a synopsis of the findings from the 2015–2020 study, which indicated that sleep variability is associated with an elevated risk of obesity, dysglycemia, type 2 diabetes, and metabolic syndrome (31, 32). Elevated sleep variability reportedly disrupts biorhythmic homeostasis, thereby diminishing anti-inflammatory capacity and potentially increasing predisposition to chronic diseases, including diabetes mellitus (33). A growing body of research suggests a potential relationship between sleep variability and the production or release of melatonin, a hormone intimately linked to glucose tolerance (34, 35). Increased sleep variability reflects alternating sleep reduction and sleep compensation, leading to circadian rhythm disruption, which may reduce insulin sensitivity and thus cause dysglycemia (31). Prolonged exposure to this phenomenon over an extended period can precipitate a cascade of adverse physiological responses, including an augmented risk of type 2 diabetes and metabolic syndrome (31). While no significant associations were observed between sleep metrics and hypertension, advanced maternal age combined with irregular sleep was linked to increased odds of hyperglycemia. Future research involving larger cohorts is essential to improve statistical power and further clarify these relationships.

The primary strengths of our study are as follows. First of all, the present study further extended previous literature by innovatively assessing the association between sleep variability and irregularity and GMS during pregnancy in a prospective birth cohort study, thereby providing new research ideas to safeguard metabolic health during pregnancy. Our study benefits from a larger, more representative sample size than comparable previous works. By tracking sleep patterns longitudinally throughout the entire pregnancy rather than at a single time point, we provide a more precise characterization of sleep dynamics during this critical period. Finally, a comprehensive set of covariates was incorporated as adjustment factors to ensure the reliability of the results and to avoid potential confounding bias. Nonetheless, several limitations must be acknowledged. First, it should be noted that in sub-cohorts 2 and 3, pre-pregnancy BMI differed significantly between included and excluded participants, with excluded participants having higher pre-pregnancy BMI. As pre-pregnancy obesity is an important risk factor for hypertension and hyperglycemia, this difference may lead to an underestimation of the associations between sleep variability and these outcomes. However, the inverse probability weighting sensitivity analyses yielded results consistent with the primary analyses, suggesting that the impact of selection bias on effect estimates may be limited. Nonetheless, the possibility of residual selection bias cannot be entirely excluded. Future studies with more complete follow-up data are warranted to validate our findings. Moreover, it must be acknowledged that self-reported sleep data is potentially subject to recall bias and exposure misclassification. Nevertheless, biases of this nature are, generally, unavoidable in large-scale prospective cohort studies. Research has shown that there is a moderate correlation between self-reported sleep assessments based on the PSQI and objectively measured sleep metrics. These findings suggest the potential utility of PSQI-based assessments in epidemiological studies (33). Although the PSQI is a validated tool widely used in epidemiological studies, it relies on self−report and recall and may not capture objective sleep parameters with the same precision as actigraphy. Self−reported sleep may be influenced by pregnancy−related changes in perception, potentially affecting the accuracy of estimates of sleep variability and irregularity. In addition, variability was estimated based on only three time points (one per trimester), which may not accurately reflect sleep fluctuations in individuals over the entire gestational period. This partial assessment could lead to non-differential misclassification of exposure and bias the associations toward the null. Consequently, our effect estimates may be conservative approximations of the true associations. Further studies incorporating more frequent sleep assessments (e.g. daily or weekly) or objective monitoring devices are required to confirm and extend our findings. Thirdly, the present study has limitations due to its single-center design and predominantly Han Chinese study population. This may restrict the generalizability of our findings to other ethnic groups or geographical regions. Differences in genetic background, cultural sleep practices (e.g. variations in sleep timing and napping habits, and social norms regarding sleep during pregnancy), socioeconomic status and healthcare systems could modify the observed associations between sleep patterns and metabolic outcomes in different populations. Therefore, further investigation is warranted to determine the generalizability of our findings to more diverse populations. Accordingly, our research team has initiated a nationwide, multicenter collaboration, and future studies with larger sample sizes and more diverse populations will further explore the relationship between sleep and metabolic health during pregnancy. Fourth, despite adjustment for a wide range of covariates, the possibility of residual confounding by unmeasured factors such as detailed dietary intake (e.g., glycemic load, macronutrient composition), objectively assessed physical activity, psychosocial stressors (e.g., work−related stress, social support), and biomarkers of inflammation or endocrine function (e.g., cortisol, inflammatory cytokines), cannot be entirely excluded. These factors may be associated with both sleep patterns and metabolic outcomes, and their omission could bias our estimates. Future studies incorporating comprehensive lifestyle and biomarker assessments are warranted to confirm and extend our findings. Finally, we acknowledge that including pre-pregnancy BMI in the GMS reflects baseline metabolic status rather than changes that occur during pregnancy. This definitional choice has implications for the interpretation of our findings. On the one hand, including pre-pregnancy BMI captures the cumulative metabolic risk spanning both the pre-conception and gestational periods. This is clinically relevant, as women who are overweight or obese when they become pregnant already carry a higher baseline metabolic burden. However, this approach may mean that our findings reflect the association between sleep variability and pre-existing metabolic status, rather than gestational metabolic changes alone. We acknowledge that alternative conceptual approaches to defining GMS exist in the literature. Some researchers have proposed definitions that focus exclusively on metabolic changes that arise during pregnancy (e.g. gestational diabetes, gestational hypertension or dyslipidaemia), without taking pre-pregnancy BMI into account. However, such definitions are not widely standardized. Our definition is based on current diagnostic criteria and is consistent with the diagnostic frameworks used in previous studies. These frameworks include pre-pregnancy BMI as a component, recognizing the cumulative metabolic risk from pre-conception through to gestation.

## 5 Conclusions

In summary, the present study demonstrates an association between sleep variability and the occurrence of GMS and dyslipidaemia. Greater sleep variability is associated with a higher likelihood of these metabolic disturbances. Exploratory subgroup analyses suggested that these associations may vary according to maternal age, conception method and gravidity; however, given the exploratory nature of these analyses and the multiple comparisons performed, these findings should be interpreted with caution and considered as generating hypotheses. Conversely, sleep irregularity was not associated with the occurrence of GMS or its components in the overall analysis. However, among pregnant women of advanced age, exploratory analyses revealed that irregular sleep patterns were associated with higher odds of hyperglycaemia. This study provides novel evidence of an association between sleep during pregnancy and GMS, which may have public health implications. Further research is necessary to extend these findings, including multicenter studies to provide additional evidence, and clinical trials to assess whether improving sleep irregularity could influence metabolic outcomes.

## Data Availability

The raw data supporting the conclusions of this article will be made available by the authors, without undue reservation.
