# Supplementary material for: Association of sleep variability and irregularity with gestational metabolic syndrome: a birth cohort study
Source: Front Endocrinol (Lausanne). 2026 May 13;17:1736419. doi: 10.3389/fendo.2026.1736419 (PMC13211974; doi:10.3389/fendo.2026.1736419)
Supplement: Supplementary file 1 [file DataSheet1.docx]

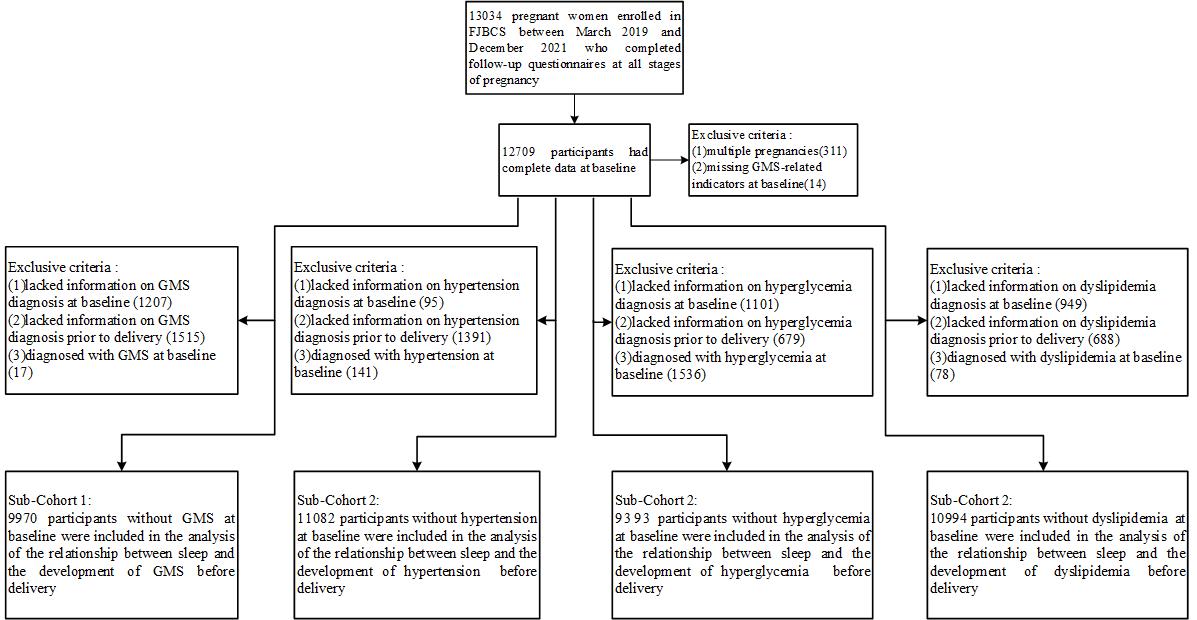


Figure S1. Study flowchart of participant selection in four sub-cohorts.

Table S1. Distribution of sleep variability and sleep irregularity measures in the study population

|  | Mean (SD) | Median (IQR) | Min-Max |
| --- | --- | --- | --- |
| Sub-Cohort 1 Occurrence of GMS | | | |
| sleep variability | 0.87(0.66) | 0.72(0.41-1.12) | 0.00-7.52 |
| sleep irregularity | 0.51(0.39) | 0.41(0.20-0.71) | 0.00-6.51 |
| Sub-Cohort 2 Occurrence of Hypertension | | | |
| sleep variability | 0.87(0.67) | 0.72(0.41-1.12) | 0.01-9.07 |
| sleep irregularity | 0.51(0.38) | 0.41(0.20-0.71) | 0.00-5.06 |
| Sub-Cohort 3 Occurrence of Hyperglycemia | | | |
| sleep variability | 0.87(0.66) | 0.72(0.41-1.12) | 0.00-7.52 |
| sleep irregularity | 0.51(0.39) | 0.41(0.20-0.71) | 0.01-6.51 |
| Sub-Cohort 4 Occurrence of Dyslipidemia | | | |
| sleep variability | 0.88(0.66) | 0.72(0.41-1.12) | 0.00-7.52 |
| sleep irregularity | 0.51(0.39) | 0.41(0.20-0.71) | 0.00-6.51 |

Abbreviations: GMS, gestational metabolic syndrome; SD, standard deviation; IQR, interquartile

range.

Table S2. Baseline characteristics of participants included and excluded in each sub-cohort

|  | Sub-Cohort 1: GMS | | | Sub-Cohort 2: Hypertension | | | Sub-Cohort 3: Hyperglycemia | | | Sub-Cohort 4: Dyslipidemia | | |
| --- | --- | --- | --- | --- | --- | --- | --- | --- | --- | --- | --- | --- |
| Variable | Included  (n=9970) | Excluded  (n=2739) | *p* | Included  (n=11082) | Excluded  (n=1627) | *p* | Included  (n=9393) | Excluded  (n=3316) | *p* | Included  (n=10994) | Excluded  (n=1715) | *p* |
| Maternal age(years)  ± mean (SD) | 30.5±3.9 | 30.6±4.1 | 0.107 | 30.5±3.9 | 30.5±4.1 | 0.957 | 30.4±3.9 | 30.8±4.2 | <0.05 | 30.5±3.9 | 30.5±4.2 | 0.550 |
| Ethnicity-Han, n (%) | 9780(98.1%) | 2688(98.1%) | 0.937 | 10872(98.1%) | 1596(98.1%) | 1.000 | 9218(98.1%) | 3250(98.1%) | 0.644 | 10984(98.1%) | 1684(98.1%) | 0.772 |
| Education, n (%)^a^ |  |  | 0.922 |  |  | 0.390 |  |  | <0.05 |  |  | 0.281 |
| Junior and below | 831(8.4%) | 224(8.2%) |  | 902(8.1%) | 153(9.4%) |  | 731(7.8%) | 324(9.8%) |  | 911(8.3%) | 144(8.4%) |  |
| High School | 1272(12.8%) | 351(12.8%) |  | 1415(12.8%) | 208(12.8%) |  | 1140(12.1%) | 483(14.6%) |  | 1380(12.6%) | 243(14.2%) |  |
| Undergraduate degree | 7143(71.7%) | 1957(71.5%) |  | 7951(71.8%) | 1149(70.7%) |  | 6813(72.6%) | 2287(69.1%) |  | 7890(71.8%) | 1210(70.6%) |  |
| Graduate and above | 713(7.2%) | 206(7.5%) |  | 803(7.3%) | 116(7.1%) |  | 701(7.5%) | 218(6.6%) |  | 802(7.3%) | 117(6.8%) |  |
| Urban-residence, N (%)^a^ | 7228(91.4%) | 2139(91.7%) | 0.704 | 8021(91.4%) | 1346(91.9%) | 0.579 | 6878(91.6%) | 2489(91.2%) | 0.603 | 8115(91.6%) | 1252(90.9%) | 0.378 |
| Pre-pregnancy BMI  (kg/m^2^), n (%) |  |  | 0.201 |  |  | <0.05 |  |  | <0.05 |  |  | 0.739 |
| Normal weight | 7528(75.5%) | 2009(74.4%) |  | 8348(75.6%) | 1189(73.2%) |  | 7059(75.4%) | 2478(74.9%) |  | 8257(75.4%) | 1280(74.7%) |  |
| Underweight | 1611(16.2%) | 438(16.2%) |  | 1800(16.3%) | 249(15.3%) |  | 1581(16.9%) | 468(14.1%) |  | 1761(16.1%) | 288(16.8%) |  |
| Overweight+ Obese | 831(8.3%) | 254(9.4%) |  | 899(8.1%) | 186(11.5%) |  | 721(7.7%) | 364(11.0%) |  | 940(8.6%) | 145(8.5%) |  |

Abbreviations: GMS, gestational metabolic syndrome; SD, standard deviation.

Table S3. Comparison of associations between sleep variability/irregularity and GMS

and its components before and after inverse probability weighting

|  | Unweighted model  aOR (95% CI)^a^ | *p* | Weighted model  aOR (95% CI)^a^ | *p* |
| --- | --- | --- | --- | --- |
| Sub-Cohort 1 Occurrence of GMS | | | | |
| sleep variability | 1.348(1.136-1.599) | **<0.001** | 1.339(1.144-1.550) | **<0.001** |
| sleep irregularity | 1.062(0.773-1.459) | 0.711 | 1.052(0.782-1.377) | 0.726 |
| Sub-Cohort 2 Occurrence of Hypertension | | | | |
| sleep variability | 1.081(0.901-1.297) | 0.403 | 1.079(0.905-1.267) | 0.375 |
| sleep irregularity | 1.102(0.800-1.517) | 0.552 | 1.089(0.802-1.450) | 0.570 |
| Sub-Cohort 3 Occurrence of Hyperglycemia | | | | |
| sleep variability | 1.020(0.947-1.098) | 0.600 | 1.032(0.969-1.098) | 0.319 |
| sleep irregularity | 1.008(0.891-1.144) | 0.897 | 1.027(0.925-1.140) | 0.617 |
| Sub-Cohort 4 Occurrence of Dyslipidemia | | | | |
| sleep variability | 1.086(1.019-1.159) | **0.012** | 1.072(1.009-1.140) | **0.024** |
| sleep irregularity | 1.069(0.959-1.193) | 0.228 | 1.038(0.938-1.149) | 0.468 |

Abbreviations: GMS, gestational metabolic syndrome; prior GDM, previous history of gestational diabetes; prior GH, previous history of gestational hypertension; aOR, adjusted relative risk; CI, confidence interval.

^a^aOR and 95% CI estimated with logistics regression adjusted for maternal age, ethnicity, educational levels and urban-residence, gravidity, parity, assisted reproduction, smoking status, alcohol consumption status, coffee consumption status, tea consumption status, average outdoor time, pre-pregnancy diabetes, pre-pregnancy hypertension, prior abnormal pregnancy, prior pregnancy complications, prior GH and prior GDM.

Table S4. Tests of interaction between sleep variability and potential effect modifiers for the association with GMS and its components

|  | GMS | | Hypertension | | Hyperglycemia | | Dyslipidemia | |
| --- | --- | --- | --- | --- | --- | --- | --- | --- |
| Variable | aOR (95% CI) ^a^ | *p* | aOR (95% CI) ^a^ | *p* | cOR (95% CI) ^a^ | *p* | cOR (95% CI) ^a^ | *p* |
| **sleep variability** |  |  |  |  |  |  |  |  |
| ×Maternal age | 1.011(1.006-1.017) | **<0.001** | 1.008(0.993-1.023) | 0.307 | 1.001(0.999-1.004) | 0.359 | 1.003(1.001-1.006) | **0.002** |
| ×Conception method | 1.275(1.090-1.490) | **0.002** | 1.365(0.949-1.965) | 0.093 | 1.017(0.974-1.063) | 0.443 | 1.069(1.009-1.132) | **0.023** |
| ×Gravidity | 1.224(1.116-1.341) | **<0.001** | 1.006(0.750-1.350) | 0.968 | 1.016(0.951-1.086) | 0.635 | 1.049(1.010-1.090) | **0.013** |
| **sleep irregularity** |  |  |  |  |  |  |  |  |
| ×Maternal age | 1.003(0.993-1.013) | 0.506 | 1.028(1.011-1.047) | **0.002** | 1.001(0.997-1.005) | 0.478 | 1.003(1.000-1.007) | 0.072 |
| ×Conception method | 1.042(0.785-1.382) | 0.778 | 2.790(1.599-4.870) | **<0.001** | 1.008(0.938-1.084) | 0.828 | 1.047(0.951-1.152) | 0.348 |
| ×Gravidity | 1.105(0.938-1.301) | 0.231 | 1.480(0.970-2.258) | 0.069 | 1.019(0.914-1.136) | 0.736 | 1.040(0.975-1.109) | 0.231 |

Abbreviations: GMS, gestational metabolic syndrome; prior GDM, previous history of gestational diabetes; prior GH, previous history of gestational hypertension; aOR, adjusted relative risk; CI, confidence interval. ^a^aOR and 95% CI estimated with logistics regression adjusted for maternal age, ethnicity, educational levels and urban-residence, gravidity, parity, assisted reproduction, smoking status, alcohol consumption status, coffee consumption status, tea consumption status, average outdoor time, pre-pregnancy diabetes, pre-pregnancy hypertension, prior abnormal pregnancy, prior pregnancy complications, prior GH and prior GDM.
